# Boosting NAD ameliorates hematopoietic impairment linked to short telomeres in vivo

**DOI:** 10.1007/s11357-023-00752-2

**Published:** 2023-02-24

**Authors:** Amanda J. Stock, Saipriya Ayyar, Amogh Kashyap, Yunong Wang, Hagai Yanai, Matthew F. Starost, Mayuri Tanaka-Yano, Monica Bodogai, Chongkui Sun, Yajun Wang, Yi Gong, Chandrakala Puligilla, Evandro F. Fang, Vilhelm A. Bohr, Yie Liu, Isabel Beerman

**Affiliations:** 1 Laboratory of Genetics and Genomics, Biomedical Research Center, National Institute On Aging/National Institutes of Health, 251 Bayview Blvd., Baltimore, MD USA; 2 Translational Gerontology Branch, Biomedical Research Center, National Institute On Aging/National Institutes of Health, 251 Bayview Blvd., Baltimore, MD USA; 3 Division of Veterinary Resources, Building 14E, National Institutes of Health, 9000 Rockville Pike, Bethesda, MD USA; 4 Laboratory of Molecular Biology and Immunology, Biomedical Research Center, National Institute On Aging/National Institutes of Health, 251 Bayview Blvd., Baltimore, MD USA; 5 DNA Repair Section, Biomedical Research Center, National Institute On Aging/National Institutes of Health, 251 Bayview Blvd., Baltimore, MD USA; 6 Department of Clinical Molecular Biology, University of Oslo and Akershus University Hospital, 1478 Lørenskog, Norway

**Keywords:** Telomere attrition/dysfunction, Telomere biology disorders, NAD metabolism, Hematopoiesis

## Abstract

Short telomeres are a defining feature of telomere biology disorders (TBDs), including dyskeratosis congenita (DC), for which there is no effective general cure. Patients with TBDs often experience bone marrow failure. NAD, an essential metabolic coenzyme, is decreased in models of DC. Herein, using telomerase reverse transcriptase null (*Tert*^−/−^) mice with critically short telomeres, we investigated the effect of NAD supplementation with the NAD precursor, nicotinamide riboside (NR), on features of health span disrupted by telomere impairment. Our results revealed that NR ameliorated body weight loss in *Tert*^*−/−*^ mice and improved telomere integrity and telomere dysfunction-induced systemic inflammation. NR supplementation also mitigated myeloid skewing of *Tert*^*−/−*^ hematopoietic stem cells. Furthermore, NR alleviated villous atrophy and inflammation in the small intestine of *Tert*^*−/−*^ transplant recipient mice. Altogether, our findings support NAD intervention as a potential therapeutic strategy to enhance aspects of health span compromised by telomere attrition.

**Supplementary Information:**

The online version contains supplementary material available at 10.1007/s11357-023-00752-2.

## Introduction

Telomeres are nucleoprotein structures that cap the ends of chromosomes from eliciting a DNA damage response [1, 2]. Telomere attrition is a major feature of aging and telomere biology disorders (TBDs), including the bone marrow failure syndromes, dyskeratosis congenita (DC) and acquired aplastic anemia [3–7]. Patients with TBDs also have an increased prevalence of pulmonary fibrosis, liver disease, cancer, and gastrointestinal pathologies, including enteropathy and enterocolitis [8]. One of the major causes of early mortality in patients with TBDs is bone marrow (BM) failure [9–11], the treatment option for which is hematopoietic stem cell or BM transplantation [9, 11, 12]. Due to complications, including organ damage, infection, and graft failure, 41% of patients with DC die within 10 years following hematopoietic stem cell transplantation [13].

Emerging evidence has suggested that cell dysfunction in patients with TBDs is linked to a critically short telomere-induced DNA damage response (DDR) [14]. Short telomeres in patients with TBDs are primarily due to germline mutations in telomere maintenance genes including the core subunits of telomerase, the telomerase RNA (*TERC*) and the telomerase reverse transcriptase (*TERT*) [3–7]. Mice deficient in *Terc* or *Tert* exhibit gradual telomere loss with successive generations (G), and at late generations (≥ G3), harbor critically short telomeres that elicit a DNA damage response and contribute to the loss of cell viability and genome stability [15–21]. Additional phenotypes in telomerase null mice that resemble those observed with aging and/or in TBD patients include decreased body weight [22–25] and hematopoietic stem cell (HSC) impairment, including decreased lymphopoiesis [26, 27], increased myelopoiesis/myeloid skewing [26], and early HSC exhaustion in serial transplantations [28, 29]. Thus, telomerase null mice serve as a disease model to evaluate interventions of HSC impairment linked to short telomeres.

Nicotinamide adenine dinucleotide (NAD), an essential metabolite in life and health, plays a fundamental role in energy production and redox homeostasis. It is also involved in a broad spectrum of cellular activities as evidenced by the association of decreased NAD to all the hallmarks of aging, including genomic instability and mitochondrial dysfunction [30, 31]. Recent findings from our group revealed that primary human fibroblasts from patients with DC as well as tissues from G3 *Tert*^*−/−*^ mice with critically short telomeres are defective in NAD metabolism, evident by decreased NAD levels, an imbalance in NAD-consuming enzyme activities, i.e. elevated CD38 NADase and reduced poly (ADP-ribose) polymerase and SIRT1 deacetylase activities, and defects in the NAD-consuming enzyme-related signaling networks, including the NAD-SIRT1-PGC-1α-mitochondria and the NAD-PARP-telomere DNA repair axis [32]. Importantly, NAD levels as well as PARP1 and SIRT1 expression/activities were restored upon treatment with the vitamin B3 analog and NAD precursor, nicotinamide riboside (NR). NR also inhibited telomere-induced DDR, improved mitochondrial morphology and parameters, and delayed replicative senescence in DC patient fibroblasts. Thus, intervention of NAD metabolism ameliorates cellular defects of DC [32]. However, it remains unclear whether NAD supplementation mitigates disease features of DC.

A decline in NAD levels has emerged as an important hallmark of aging, including stem cell exhaustion, while supplementation with NAD precursors improved NAD levels and health span in aged and DNA repair-deficient animal models with premature aging phenotypes [30, 31, 33–37]. Recently, our group has revealed beneficial effects of NR on HSC lymphoid potential in aged and in ataxia-telangiectasia mutated kinase (ATM) DNA repair-deficient mice [38], supporting the notion that NR could benefit HSC function in critical conditions associated with impaired hematopoiesis. Additionally, other groups have demonstrated benefits of NR on HSCs, including improved lineage potential [39]. In this study, we employed late-generation *Tert*^*−/−*^ mice to determine the effects of NR supplementation on vital aspects of health span that are compromised due to short telomeres. We found that late-generation *Tert*^*−/−*^ mice displayed significant impairments in hematopoiesis, with a decreased lymphoid/myeloid ratio in their blood composition. Importantly, NR boosted NAD levels and significantly improved B-cell output and telomere integrity, as well as mitigated systemic inflammation of late-generation *Tert*^*−/−*^ mice in a resting and/or BM transplant setting. Our findings illuminate mechanisms underlying the benefits of NR in the context of telomere erosion and advance progress in developing therapeutic strategies for TBDs.

## Methods

### Animals and treatment

*Tert*^*−/−*^ mice (C57BL/6 background) [16] were administrated with 12 mM NR (Niagen®, Chromadex) in their drinking water for 2–10 months for resting-state analyses. *Tert*^*−/−*^ mice (CD45.2) were treated for 10 months prior to resting-state analysis of *Tert*^*−/−*^ BM (at age 13 months) and transplantation of *Tert*^*−/−*^ BM into B6.SJL-Ptprca Pepcb/BoyJ (CD45.1, Jackson Laboratory) recipients. *Tert*^*−/−*^ transplant recipient mice were treated for 1 month prior to transplantation and during the 4-month post-transplant follow-up period. Body weights were recorded monthly for mice treated for 10 months. All animal experiments were carried out according to the ‘‘Guide for the Care and Use of Laboratory Animals’’ (National Academy Press, USA, 1996), and were approved by the Institutional Animal Care and Use Committee of the National Institute on Aging (ASP #383-LGG-2025 and 469-TGB-2025).

### Bone marrow transplantation

For experiments assessing the cell-intrinsic effects of NR, 1.34 × 10^6^ whole bone marrow (WBM) cells from 13-month-old vehicle- or NR-treated *Tert*^*−/−*^ mice (CD45.2) were competitively transplanted against 6.6 × 10^5^ CD45.1 WBM cells in 10 lethally irradiated (9.56 Gy) 2.5-month-old CD45.1 recipients by tail vein injection. For experiments assessing the cell-extrinsic effects of NR, 2 × 10^6^ WBM CD45.1 donor cells were transplanted into 2–5 month-old vehicle-treated G1 *Tert*^*−/−*^ recipients, vehicle-treated G3 *Tert*^*−/−*^ recipients, and NR-treated recipients.

### Peripheral blood analysis

Peripheral blood (PB) was retro-orbitally collected and analyzed for donor chimerism and lineage contribution by FACS, and complete blood cell counts (CBC) were measured using a Hemavet 950FS. PB cells were treated with ammonium-chloride-potassium solution (ACK) and stained with Ter119-PerCP Cy5.5 (Biolegend # 116,227), B220-APC/Cy7 (Biolegend # 103,223), Mac1-PeCy7 (Biolegend # 101,215), CD3-PE (Biolegend # 100,205), and Gr1-FITC (Biolegend # 108,406). CD45.1-APC (Biolegend # 110,714) and CD45.2-Pacific Blue (Biolegend # 109,820) were included in the analysis of transplanted mice. Following the 45-min staining, cells were washed with 1 × PBS and resuspended in 1 µg/ml propidium iodide (PI) (ThermoFisher Scientific). Analysis was performed using a BD FACS Canto II flow cytometer and FlowJo Prism software (Becton Dickinson). In experiments utilizing *Tert*^*−/−*^ mice as transplant recipients, all mice were alive at 4 weeks post-transplant and analyzed as one cohort, while mice treated for 8 weeks or 16 weeks were divided into 2 different cohorts due to their different end points. One G1 *Tert*^*−/−*^ mouse was excluded from CBC analysis at 16 weeks post-transplant due to insufficient cell numbers.

### FACS isolation of cells

HSC sorting was performed as previously described [38]. For sorting of granulocytes, PB was treated with ACK and stained using the same protocol and antibody cocktail described above, with the exception of anti-Gr1, which was conjugated to brilliant violet 510 (BV510, Biolegend # 108,438). Cells were analyzed and sorted using a BD FACS Fusion flow cytometer.

### Whole bone marrow analysis

Bone marrow (BM) cells were obtained by crushing long bones of the mice and treated with ACK, washed with 1X PBS, and stained with antibody cocktails. For non-transplanted mice, BM was stained with antibodies against lineage surface markers conjugated to biotin (Ter119, Biolegend # 116,204, 1/200; B220, Biolegend # 103,204, 1/200; Mac1, Biolegend # 101,204, 1/200; CD3, Biolegend # 100,244, 1/200; Gr1, Biolegend #108,404 1/200), ckit-PE (Biolegend # 105,808, 1/200), Sca1 conjugated to APC/Cy7 (Biolegend # 108,126, 1/200), CD34-FITC (eBioscience # 11–0341-85, 1/50), Flk2 -APC (Biolegend # 135,310, 1/50), CD150-PE/Cy7 (Biolegend # 115,914, 1/200), IL-7Rα-BV421 (Biolegend # 135,024, 1/100), and FcγRα-PerCP/Cy5.5 (eBioscience # 45–016-82, 1/100). After a 1.5 h incubation, cells were washed and incubated with streptavidin-Pacific Orange (ThermoFisher Scientific) for 30 min. Cells were washed in sample media (1X PBS, 2% FBS, 2 mM EDTA) and resuspended in 1 µg/mL PI solution and analyzed on a BD FACSAria Fusion. BM was stained using a panel of antibodies for detecting primitive stem cells and multipotent progenitors: lineage, c-Kit, Sca1, CD34, Flk2, CD150, and FcγRα (as above), CD45.1-BV711 (BD # 747,742, 1/100), CD45.2-Pacific Blue (anti-109820, 1/100), and CD48 -BV786 (BD #740,876). Lymphoid progenitors were assayed with a separate stain: lineage (without anti-B220), Flk2, CD45.1, CD45.2 (as above), CD27-PE (eBioscience # 12–0271-83 1/200), IL-7Rα- FITC (Biolegend # 135,008), B220-APC/Cy7 (Biolegend # 103,224), Ly6d conjugated to PerCP, and PI. Samples were analyzed on a BD FACSAria Fusion. Technical errors with the flow cytometer resulted in an inadequate sample size and inability to collect data for 3 of the NR-treated samples in the panel used for detecting HSCs and CLPs.

*Tert*^*−/−*^ recipients were euthanized at 8 and 16 weeks post-transplant and stained with the same antibodies used above for lineage (without anti-B220), IL-7Rα-BV421 (as above), B220-APC/Cy7 (Biolegend # 103,224), CD45.1-PE (ThermoFisher # 61–0453-82, 1/100), CD45.2-BV711 (Biolegend # 109,847), and PI. Samples were analyzed on a BD FACSymphony A3. FlowJo (Treestar) and Cytobank (Beckman Coulter) software were used to analyze the data.

### Serum and bone marrow cytokine assays

For serum, PB was collected into BD microtainer serum tubes (VWR) and allowed to clot for 30 min. Samples were centrifuged at 1500 × g for 10 min at 4 °C to remove clotting factors. Samples were diluted (1:1) with PBS, pH 7.5 and submitted to Eve Technologies (Calgary, AB Canada) using the mouse 31-plex array (#MD31). Three cytokines were excluded from the analysis due to technical errors, and one cytokine (IL-3) was excluded from the display only (but not statistical analysis) due to extremely high variability among mice. For BM, samples were briefly treated with ACK, washed in PBS, and cryopreserved in 90% FBS and 10% dimethyl sulfoxide in liquid nitrogen. Cells were restored, spun, and washed in PBS prior to resuspension in ice-cold PBS containing 0.1% IGEPAL CA-630 (Sigma-Aldrich) and Halt protease inhibitor cocktail (ThermoFisher Scientific) for 10 min. Samples were homogenized with a needle and syringe (21-gauge needle, 15 pumps), followed by centrifugation at 16,000 × g for 10 min at 4 °C, and the supernatants were stored at – 80 °C prior to shipping. BM samples were normalized to 5 µg/ml prior to multiplex analysis (#MD31) by Eve Technologies. Nine cytokines could not be detected, and two cytokines were excluded due to technical errors. Serum cytokine analysis was excluded in a couple of G3 *Tert*^*−/−*^ mice as well as all vehicle-treated G4 *Tert*^*−/−*^ mice, due to rapid decline in their health.

### Indirect immunofluorescence and telomere fluorescence in situ hybridization

Telomere dysfunction-induced foci were detected following similar methodology as our previous study [32] with minor modifications. Isolated granulocytes and BM cells were spun onto poly-l-lysine-coated cytology spin slides (Azer Scientific, Morgantown, PA). Cells were immediately fixed in ice-cold methanol at – 20 °C for 10 min, followed by 2 washes in 1 × PBS at room temperature (RT). For detecting TIFs in granulocytes, cells were permeabilized with CSK solution (10 mM PIPES, pH 6.8, 100 mM NaCl, 300 mM sucrose, 3 mM MgCl_2_, 0.5% Triton X-100) for 5 min on ice twice. Cells were washed in 1 × PBS and blocked in 3% BSA for 1 h at RT, followed by denaturation at 80 °C for 3 min and hybridization with a probe specific for telomeric DNA conjugated to Cy3 (Tel-C-Cy3 probe, PNA Bio) for 2 h at RT. Slides were then washed in hybridization wash buffer (70% formamide in 10 mM 1 M Tris–HCl, pH 7.5), followed by 1 × PBS with 0.1% tween. Cells incubated with anti-53BP1 (1:1000, Novus Biologicals # NB100-304) overnight at 4 °C protected from light. For detecting proliferating HSCs, cells were washed with 1 × PBS, permeabilized with 0.2% Triton X-100 for 5 min, blocked in 3% BSA for 1 h at RT, and incubated with anti-Ki67 (1:1,000, Abcam # ab15580). After incubation with primary antibodies, granulocytes, and HSCs were washed in 1 × PBS, incubated with Alexa fluor 488-conjugated donkey anti-rabbit secondary antibody (1:500, ThermoFisher Scientific) for 1 h at RT, washed in 1 × PBS, mounted with DAPI, and cover-slipped. Images were captured on a Zeiss Axio Observer microscope. TIFs were scored based on the colocalization of 53BP1 with telomere FISH signals. Quantification of the number of TIFs per cell as well as the percentage of cells with ≥ 5 TIFs per genotype was calculated.

### Detection of mRNA

Quantitative reverse transcriptase polymerase chain reaction (qPCR) was used to determine mRNA expression of the inflammatory cytokines, IL-6 and G-CSF, in small intestines and whole BM as previously described [40]. Primers were purchased from Integrated DNA technologies, and the sequences are as follows:

- *G-CSF* forward: 5′ -CCAGAGGCGCATGAAGCTAAT- 3′
- *G-CSF* reverse: 5′ -CGGCCTCTCGTCCTGAACAT- 3′
- *Il-6* forward: 5′ -GAAGTTCCTCTCTGCAAGAGAC -3′
- *Il-6* reverse: 5′ -GCCTCCGACTTGTGAAGTGG -3′

### NAD measurement

NAD levels were measured using the NAD kit from Abcam (# ab65348) following the manufacturer’s recommendations.

### Statistical analysis

Statistical analysis was performed using GraphPad Prism 9.0 software. All data are expressed as mean ± SEM. Individual pairs were assessed using Student’s unpaired *t*-tests, and analysis with more than 2 groups was assessed by ANOVAs. Details on post hoc analyses that were performed can be found in the figure legends. *P* values < 0.05 were considered significant.

## Results

### NAD supplementation with NR ameliorates body weight loss and improves telomere integrity in late-generation telomerase null mice

**Fig. 1:**
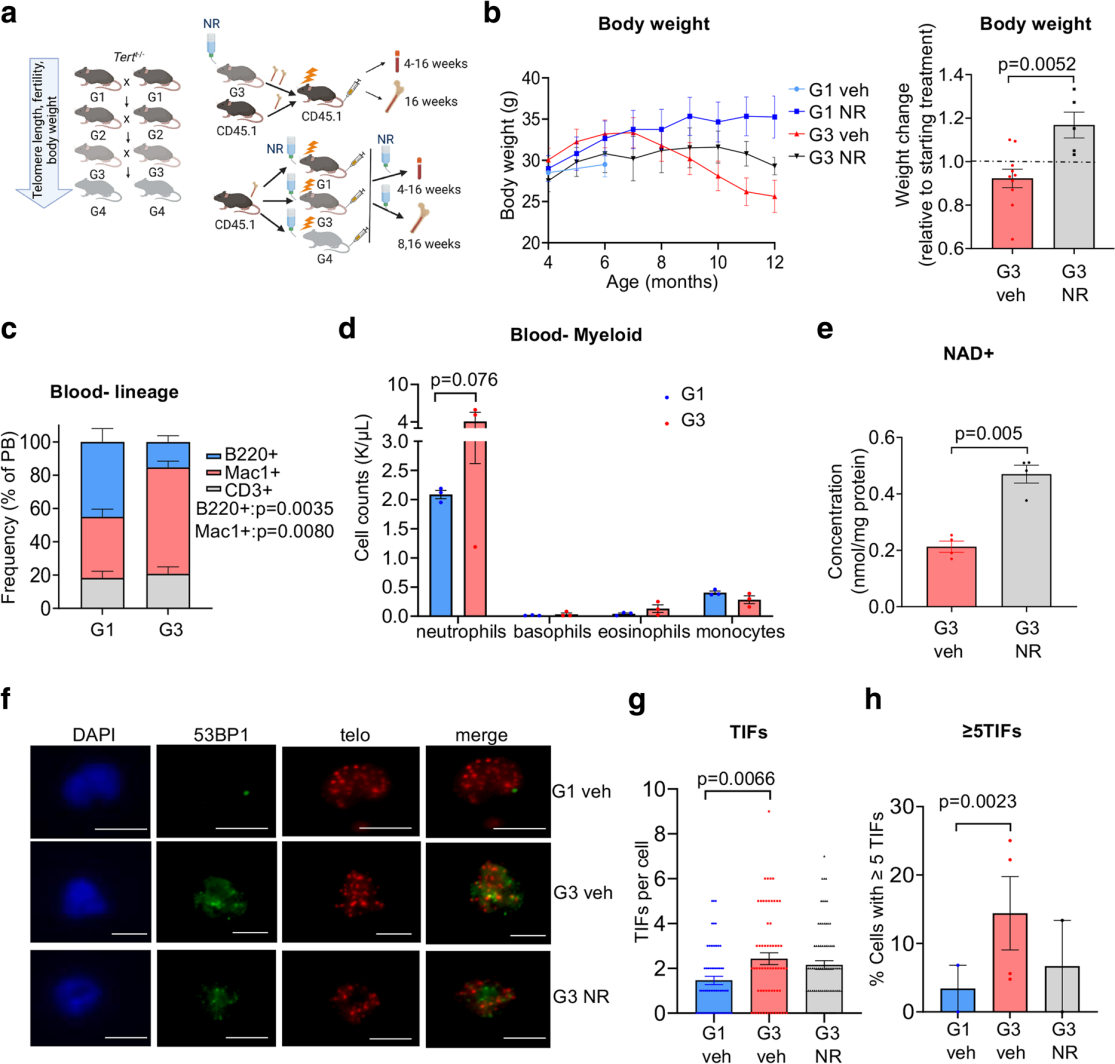
NR treatment improves resting-state body weight and telomere integrity in G3 *Tert*^*−/−*^ mice. **a** Schematic illustrating accruing deficits with successive generational interbreeding of *Tert*^*−/−*^ mice as well as transplantation synopsis (created with BioRender.com). BM from vehicle- and NR-treated G3 *Tert*^*−/−*^ (CD45.2) mice was competitively transplanted against CD45.1 BM at a 2:1 ratio into lethally irradiated CD45.1 recipients by tail vein injection. Blood was analyzed at 4–16 weeks and BM at 16 weeks post-transplant. **b** Line graph shows the absolute body weights in grams (g) of vehicle- and NR-treated mice over time. Bar graph shows the net change in body weight relative to the initiation of vehicle or NR treatment. G1 vehicle-treated (light blue, *n* = 3), G1 NR-treated (blue, *n* = 6), G3 vehicle-treated (red *n* = 10), and G3 NR-treated (gray, *n* = 5). **c** Flow cytometry analysis showing lineage contribution of live B cells (B220^+^, blue), myeloid cells (Mac1^+^, red), and T cells (CD3^+^, gray) in G1 (*n* = 3) and G3 (*n* = 4) *Tert*^*−/−*^ mice. **d** CBC analysis showing myeloid cell counts in thousands (K)/µL in G1 (blue, *n* = 3) and G3 (red, n = 3) *Tert*^*−/−*^ mouse peripheral blood. **e** Intracellular NAD levels in bone marrow-derived from vehicle-treated or NR-treated G3 *Tert*^*−/−*^ mice. N = 4 per group. **f** Representative TIFs in granulocytes sorted from mouse peripheral blood of G1 vehicle- (*n* = 2), G3 vehicle- (*n* = 4), or NR-treated (*n* = 2) *Tert*^*−/−*^ mice showing colocalization of telomere DNA (red) and 53BP1 (green) by IF-Telomere FISH. Scale bars, 10 µm. **g** Quantification of the number of TIFs per granulocyte in each group. ~ 70 cells/group were counted. Each data point represents an individual cell. **h** Quantification of the percentage of granulocytes with ≥ 5 TIFs. Each data point represents an individual mouse. *P* values were determined by student’s unpaired *t*-tests in **b** and **e**, by two-way ANOVAs with Sidak’s multiple comparisons in **c** and **d**, by a one-way ANOVA with Tukey’s multiple comparisons in **g**, and by Fisher’s exact test in **h**. *P* values in the figure are not significant unless indicated. All data are mean ± SEM

To determine the effects of NR on aspects of health span impaired by telomere erosion, we utilized a mouse strain deficient in telomerase reverse transcriptase (*Tert*^−/−^), which exhibits progressive loss of telomere length, fertility, and body weight in successive generations (G) [16, 22, 23, 41] (Fig. 1a). Similarly, developmental delays and a failure to thrive occur in many cases of human patients with TBDs [11]. Oral NR supplementation of G3 *Tert*^*−/−*^ mice after weaning led to a net increase in body weight (Fig. 1b), supporting the concept that NR supplementation provides a beneficial effect on mice with eroded telomeres.

Patients with TBDs exhibit severe deficits in hematopoiesis, resulting in bone marrow failure [9–11]. G3 *Tert*^*−/−*^ mice also showed impairments in hematopoiesis, including a trend for a decreased frequency of common lymphoid progenitors in the bone marrow (BM) (Fig S1a) and a decreased B-lymphoid/myeloid ratio in peripheral blood (PB) (Fig. 1c), with a strong trend for an increased neutrophil frequency compared to G1 *Tert*^*−/−*^ mice (Fig. 1d). NAD + levels were similar between G3 and G1 *Tert*^*−/−*^ bone marrow at age 6 months (Fig S1c). This finding is consistent with results from a previous study [42] that detected no difference in NAD + levels in mouse liver tissues from late-generation *Tert*^*−/−*^ compared to WT mice, yet still revealed ameliorated liver fibrosis when these mice were supplemented with a NAD + precursor. Upon observing various benefits of NR in primary fibroblasts from DC patients [43], we supplemented G3 *Tert*^*−/−*^ mice orally with NR or vehicle control for 2 and 10 months. In a resting state, NR treatment significantly enhanced NAD levels in the bone marrow (Fig. 1e) but did not significantly affect the frequencies of primitive stem/committed progenitors within the BM (Fig S1a-b), nor PB cell compositions (Fig S1d). Within the primitive hematopoietic compartment, NR treatment on G3 *Tert*^*−/−*^ mice did result in a trend for increased frequency of HSCs (Fig S1a-b). This increased HSC frequency was not attributed to increased proliferation, as immunofluorescent staining with anti-Ki67 did not show significant changes in cell cycling of G3 *Tert*^*−/−*^ HSCs with NR treatment (Fig S1e-f). Collectively, our data suggest that long-term NR treatment may increase the survival of HSCs with critically short telomeres.

Mounting evidence has suggested that cell dysfunction in patients with DC is primarily driven by a critically short telomere-induced DNA damage response [14]. Similarly, myeloid progenitor cells from late-generation *Tert*^*−/−*^ mice expressed significantly higher levels of the DNA damage markers compared to wild type [22]. In agreement with these observations, G3 *Tert*^*−/−*^ granulocytes showed increased numbers of telomere dysfunction-induced DNA damage foci (TIFs), measured by colocalization of a DNA damage response protein, 53BP1 and telomeric DNA (Fig. 1f, g) and an increased percentage of cells with ≥ 5 TIFs (Fig. 1h), compared to G1 *Tert*^*−/−*^ granulocytes by indirect immunofluorescence and telomere fluorescence in situ hybridization (IF telomere-FISH), and NR treatment led to a trend for a decreased percentage of granulocytes with ≥ 5 TIFs (Fig. 1f–h). Our data indicate that NR helps maintain telomere integrity in G3 *Tert*^*−/−*^ mice.

### NAD supplementation with NR improves telomere integrity and increases B-lymphoid composition of late-generation telomerase null mice in a BM transplant setting

Impairments in hematopoiesis in late-generation telomerase null mice become apparent in a challenged setting [25, 27, 29, 44]. Although NR treatment increased NAD in the bone marrow of G3 *Tert*^*−/−*^ mice, NR might induce cell-extrinsic changes that would only be apparent in a bone marrow transplant setting. To test this, bone marrow pooled from vehicle- or NR-treated G3 *Tert*^*−/−*^ mice was competitively transplanted into lethally irradiated wild-type (WT) congenic recipients at a 2:1 whole bone marrow ratio (Fig. 1a). BM analysis of NR-treated compared to vehicle-treated G3 *Tert*^*−/−*^ transplant recipients did not display any differences in donor chimerism (Fig S2a) or frequency of stem cells (HSC) or lineage-committed (myeloid and lymphoid) progenitor cells (Fig S2b); however, NR treatment significantly decreased TIFs as well as the percentage of BM cells with ≥ 5 TIFs (Fig. S3) by IF telomere-FISH analysis.

**Fig. 2:**
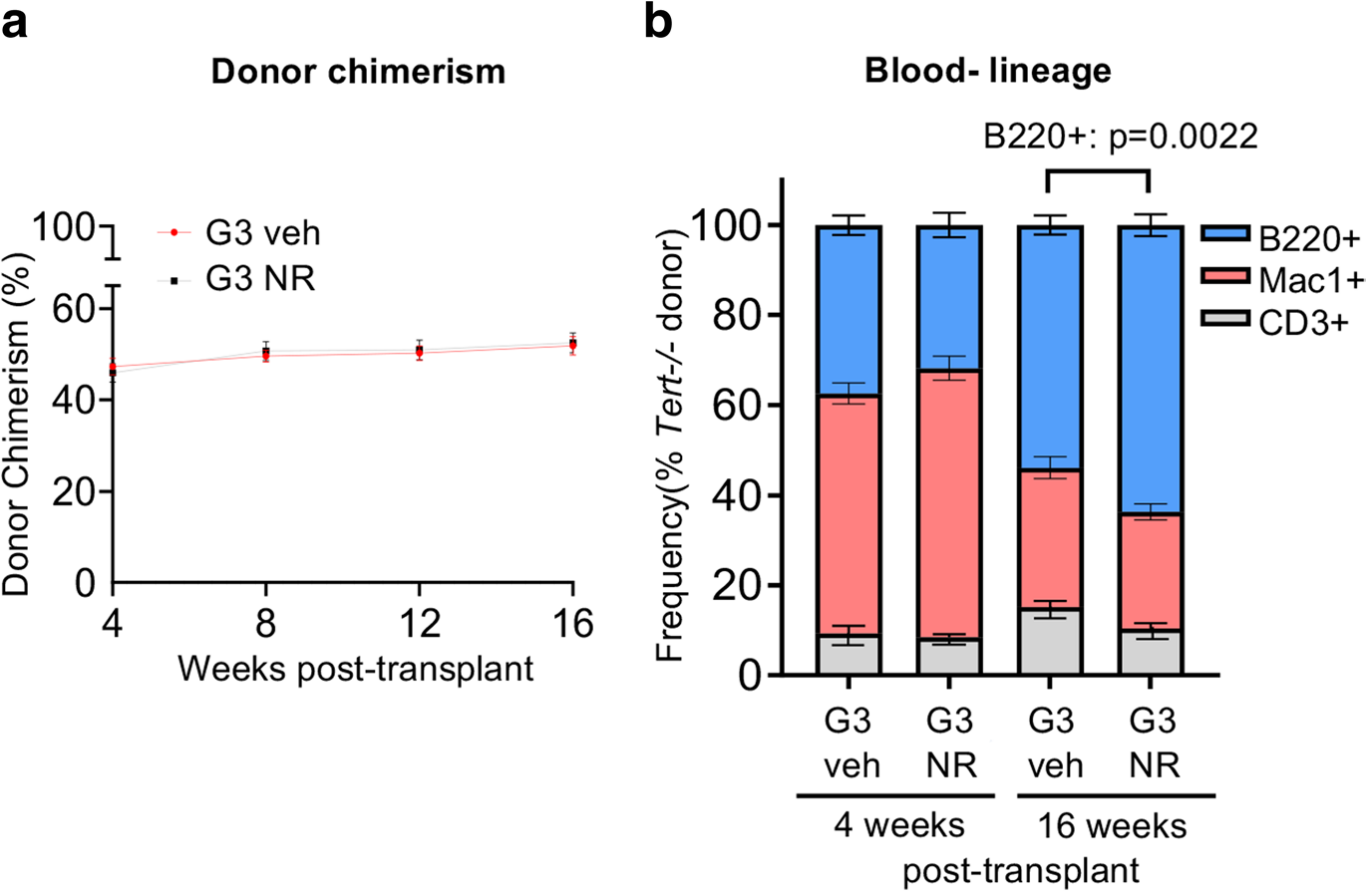
NR improves B-lymphoid composition in a BM transplant setting. **a** Vehicle and NR-treated *Tert*^*−/−*^ donor chimerism of peripheral blood in transplant recipients at 4–16 weeks post-transplant. *n* = 4 donor (pooled) and *n* = 10 recipient mice per group. **b** Flow cytometry analysis of lineage contribution of B cells (B220^+^, blue), myeloid cells (Mac1^+^, red), and T cells (CD3^+^, gray) in *Tert*^*−/−*^ donor-derived peripheral blood cells at 4 and 16 weeks post-transplant. *n* = 4 donor (pooled) and *n* = 10 recipient mice per group. *P* values were determined by two-way ANOVA with Sidak’s multiple comparisons. *P* values in the figure are not significant unless indicated. Data are mean ± SEM

Next, we assessed donor chimerism and lineage contribution in the peripheral blood of recipients every 4 weeks for 16 weeks post-transplant by flow cytometry. No differences in blood donor chimerism were observed in the vehicle- compared to NR-treated G3 *Tert*^*−/−*^ mice (Fig. 2a). PB composition was unaffected by NR at 4 weeks post-transplant (Fig. 2b), but at 16 weeks post-transplant, when the system should have returned to homeostasis, a significant increase in donor-derived B-lymphoid contribution was observed from BM cells from NR-treated compared to vehicle-treated G3 *Tert*^*−/−*^ mice (Fig. 2b). Collectively, these results support the premise that NR preserves telomere integrity of G3 *Tert*^*−/−*^ BM cells and contributes to the increased B-lymphoid contribution in transplanted recipients.

### NAD supplementation with NR prevents myeloid skewing and neutrophilia in late-generation telomerase null mice in a BM transplant setting

Previous studies have suggested that decreased lymphopoiesis in late-generation telomerase null mice is primarily due to cell-extrinsic effects of the systemic macroenvironment [26, 27]. To address the possibility that NR could affect late-generation *Tert*^*−/−*^ hematopoiesis in a cell-extrinsic manner, BM pooled from WT donor mice (CD45.1) was non-competitively transplanted into lethally irradiated G1, G3, and G4 *Tert*^*−/−*^ recipients treated with vehicle or NR (Fig. 1a). As expected, complete donor chimerism was observed in the PB (Fig S4a) and BM (data not shown) in all groups.

**Fig. 3:**
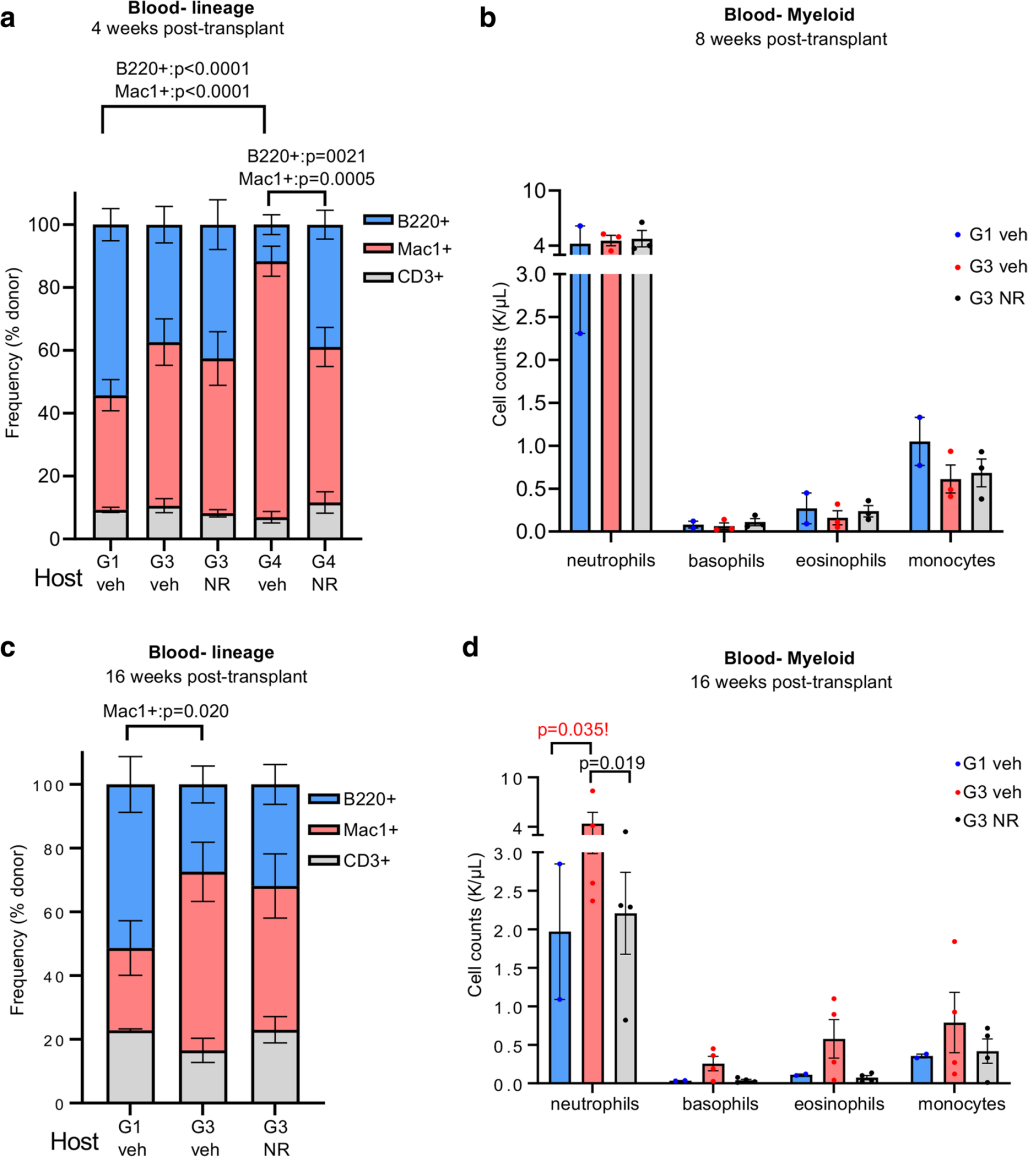
NR treatment ameliorates myeloid skewing and neutrophilia in a BM transplant setting. **a**, **c** Flow cytometry analysis of percentage lineage contribution of donor-derived peripheral blood B cells (B220 + , blue), myeloid cells (Mac1 + , red), and T cells (CD3 + , gray) in vehicle- or NR-treated *Tert*^*−/−*^ transplant recipients at 4 and 16 weeks post-transplant. *n* = 5, 9, 3, 4 for G1 veh, G3 veh, G4 veh, and G4 NR at 4 weeks. *n* = 3, 4, 4 for G1 veh, G3 veh, and G3 NR at 16 weeks. **b**, **d** CBC analysis of myeloid cell counts [in thousands (K)/µL] in *Tert*^*−/−*^ transplant recipients’ blood at 8 and 16 weeks post-transplant. *n* = 2, 3, 3 for G1 veh, G3 veh and G3 NR at 8 weeks post-transplant (**b**) and *n* = 2, 4, 4 for G1 veh, G3 veh and G3 NR at 16 weeks post-transplant (**d**). *P* values were determined by two-way ANOVAs with Sidak’s multiple comparisons in a,c and Tukey’s multiple comparisons in b,d. *P* values in the figure are not significant unless indicated. *P* value in red with exclamation mark is based on few samples. Thus, the difference shown is not statistically significant. All data are mean ± SEM. Mice were treated with vehicle or NR for 1 month prior to transplantation

At 4 weeks post-transplant, we observed a significantly elevated myeloid composition and a significantly lower B-lymphoid composition in G4 compared to G1 *Tert*^*−/−*^ recipients (Fig. 3a). Furthermore, NR treatment significantly increased B-cell and decreased myeloid composition in G4 *Tert*^*−/−*^ recipients (Fig. 3a). Compared to G1 *Tert*^*−/−*^ recipients, G3 *Tert*^*−/−*^ recipients exhibited a trend for increased myeloid composition, which was modestly mitigated with NR treatment (Fig. 3a).

Although the majority of *Tert*^*−/−*^ recipients were able to survive to 16 weeks post-transplant, a significant number of animals started to physically decline rapidly at 6 weeks post-transplant. All G4 vehicle-treated mice required euthanasia prior to 8 weeks post-transplant. At 16 weeks post-transplant, all remaining mice were euthanized for blood and bone marrow analysis. Consistent with our observations in treated G3 *Tert*^*−/−*^ mice, NR did not affect frequencies of CLPs or HSCs within the BM of G3 *Tert*^*−/−*^ recipients (Fig S4b). However, donor-derived myeloid progenitors in the BM were significantly decreased in G3 compared to G1 *Tert*^*−/−*^ mice, and NR treatment prevented the decline in BM myeloid progenitors (Fig S4b).

At 8 weeks post-transplant, no differences in PB cell composition were detected between vehicle and NR-treated G3 *Tert*^*−/−*^ recipients by flow cytometry (data not shown) or complete blood count (CBC) analysis between vehicle or NR-treated G3 *Tert*^*−/−*^ recipients (Fig. 3b). At 16 weeks post-transplant a higher myeloid contribution was observed in G3 compared to G1 *Tert*^*−/−*^ recipients (Fig. 3c). NR treatment led to a trend for decreased myeloid composition and increased lymphoid composition in G3 *Tert*^*−/−*^ recipients (Fig. 3c). CBC analysis revealed neutrophilia in G3 compared to G1 *Tert*^*−/−*^ recipients, which was significantly ameliorated by NR (Fig. 3d). Collectively, our results indicate that NR improves hematopoiesis in transplanted recipients through a non-cell-intrinsic manner.

### NAD supplementation ameliorates elevated G-CSF in late-generation telomerase null transplant recipients

**Fig. 4:**
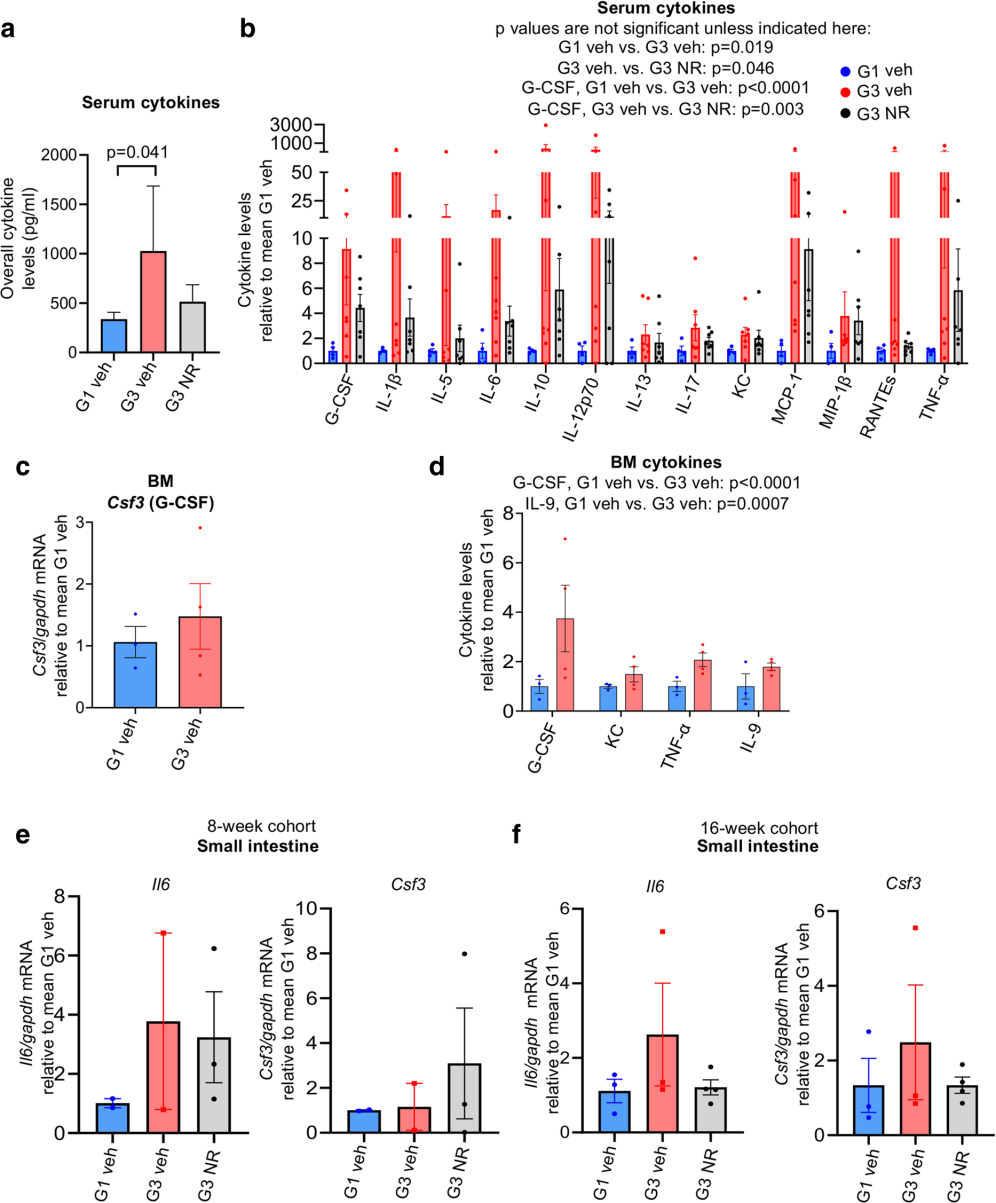
NR ameliorates elevated G-CSF in G3 *Tert*^*−/−*^ transplant recipients. **a** The overall levels of cytokines and chemokines in serum of G1 vehicle-, G3 vehicle- and NR-treated *Tert*^*−/−*^ recipients. **b** Individual serum cytokine levels in G3 vehicle- and NR-treated *Tert*^*−/−*^ recipients, relative to mean cytokine expression in G1 vehicle-treated *Tert*^*−/−*^ recipients. n = 4, 7, 7 for G1 veh, G3 veh, G3 NR. **c** mRNA expression of *Csf3* (G-CSF) in the BM of vehicle- and NR-treated G3 *Tert*^*−/−*^ recipients relative to the mean G-CSF expression in G1 vehicle-treated *Tert*^*−/−*^ mice. **d** Individual cytokine levels in vehicle-treated G3 compared to G1 *Tert*^*−/−*^ BM. **e**–**f** *Il6* (G-CSF) and *Csf3* (G-CSF) mRNA expression in the small intestines of G1 vehicle-, G3 vehicle-, and G3 NR-treated mice at 8 weeks (**e**) and 16 weeks (**f**) post-transplant. *P* values were determined using two-way ANOVA’s with Tukey’s multiple comparisons in **a**, **b**, **d**, by a student’s unpaired *t*-test in **c**, and by one-way ANOVAs in **e**–**f**. *P* values in the figure are not significant unless indicated. All data are mean ± SEM

Because hematopoiesis is influenced not only by the stem cell microenvironment, but also by the systemic macroenvironment, which includes circulating growth factors and cytokines [26, 45], we posited that the increased myeloid composition in late-generation *Tert*^*−/−*^ transplant recipients could be accompanied by increased myeloid-driven systemic inflammation. Since NR has been found to reduce inflammation in humans [46, 47] and mice [38, 48] with aging and disease, we tested whether NR ameliorates myeloid skewing in late-generation *Tert*^*−/−*^ transplant recipients, in part, by modulating inflammatory cytokines. Multiplex cytokine array analysis demonstrated upregulated levels of pro- and anti-inflammatory cytokines in the serum of G3 compared to G1 *Tert*^*−/−*^ recipients (Fig. 4a, Supplementary Table 1). NR treatment led to a trend for reduced overall cytokine expression in G3 *Tert*^*−/−*^ recipients (Fig. 4a, Supplementary Table 1). Pro-inflammatory cytokines, such as IL-6, MCP-1, and TNF-α, as well as IL-1β (known to promote myeloid differentiation [49]) and the anti-inflammatory cytokine, IL-10, were elevated in G3 compared to G1 *Tert*^*−/−*^ recipients, but showed a trend for decreased expression with NR supplementation (Fig. 4b, Supplementary Table 1). Few cytokines were either not affected by NR or not increased in G3 relative to G1 *Tert*^*−/−*^ recipients (Supplementary Table 1). Importantly, granulocyte colony-stimulating factor (G-CSF), a growth factor that stimulates the bone marrow to produce neutrophils [50], was significantly increased in G3 compared to G1 *Tert*^*−/−*^ recipient mice. Moreover, NR significantly ameliorated the elevated levels of G-CSF in G3 *Tert*^*−/−*^ recipients (Fig. 4b, Supplementary Table 1). These findings are consistent with the neutrophilia observed in G3 *Tert*^*−/−*^ recipients, which was alleviated by NR (Fig. 3d). To determine if the increase in G-CSF and other cytokine levels were derived from the BM, a subset of BM samples were assessed for cytokine levels by multiplex and/or quantitative reverse transcription-polymerase chain reaction (qPCR) analysis. G-CSF mRNA expression was slightly elevated (Fig. 4c), and G-CSF cytokine levels were significantly increased in G3 compared to G1 *Tert*^*−/−*^ BM (Fig. 4d, Supplementary Table 2). A significant increase in the pro-inflammatory cytokine IL-9 and a trend for increased levels of pro-inflammatory cytokines, KC and TNF-α, were also detected in the BM (Fig. 4d, Supplementary Table 2). Therefore, the G3 *Tert*^*−/−*^ bone marrow microenvironment likely facilitated increased production of cytokines, including G-CSF. Other than IL-9, NR did not ameliorate expression of these pro-inflammatory cytokines in the bone marrow (Supplementary Table 2), indicating that NR treatment may exert more anti-inflammatory benefits in the systemic macroenvironment than the bone marrow microenvironment. Supporting this notion, analysis of the small intestines from a subset of *Tert*^*−/−*^ mice also revealed slightly increased mRNA expression of the pro-inflammatory cytokines, IL-6 and G-CSF, in G3 compared to G1 *Tert*^*−/−*^ mice (Fig. 4e, f), which were modestly ameliorated with NR at 16 weeks post-transplant (Fig. 4f, results did not reach statistical significance). Collectively, our results support that boosting NAD by NR prevents myeloid skewing and neutrophilia in late-generation *Tert*^*−/−*^ mice in a non-cell autonomous manner, potentially by improving the systemic macroenvironment and ameliorating elevated G-CSF.

### NR ameliorates enteritis and improves survival in late-generation telomerase null mice post-irradiation

**Fig. 5:**
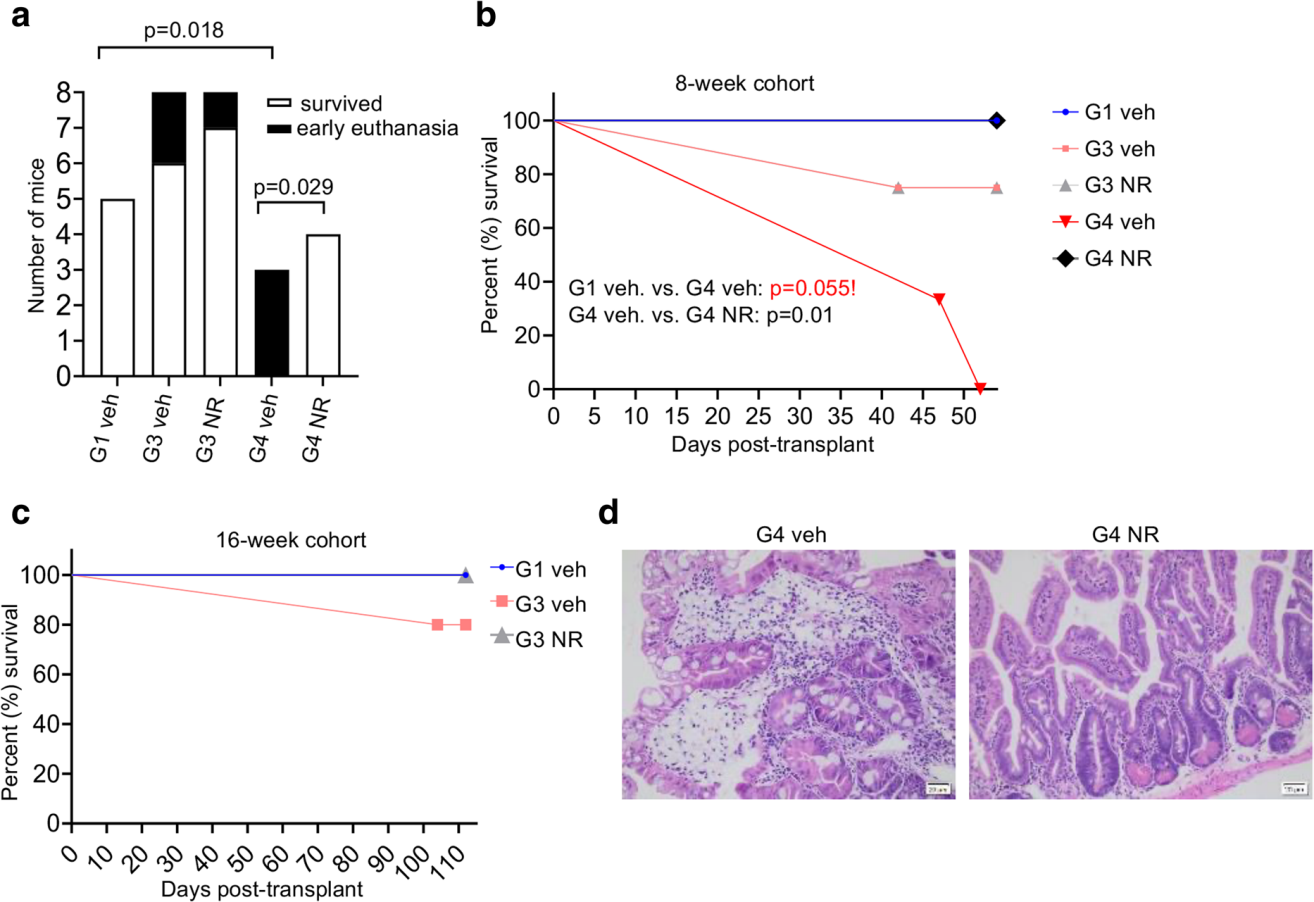
NR improves survival and ameliorates enteritis in late-generation *Tert*^*−/−*^ mice post-irradiation. **a** The distribution of mice that survived until endpoint collection or required early euthanasia. *P* values were determined by Fisher’s exact tests. **b**, **c** Kaplan–Meier survival curves of *Tert*^*−/−*^ transplant recipients that were euthanized at 8 or 16 weeks post-transplant. *n* = 2, 3, 3, 3, 4 for G1 veh, G3 veh, G3 NR, G4 veh, and G4 NR at 8 weeks (54 days), and *n* = 3, 5, 4 for G1 veh, G3 veh, and G3 NR at 16 weeks (112 days). *P* values were determined by Log-rank (Mantel-Cox) tests. **d** Representative H&E stains of small intestines exhibiting enteritis from vehicle- and NR-treated G4 *Tert*^*−/−*^ mice. Vehicle-treated G4 *Tert*^*−/−*^ mice exhibited more severe enteritis than NR-treated G4 *Tert*^*−/−*^ mice. *P* values in the figure are not significant unless indicated. *P* value in red with exclamation mark is based on few samples. Thus, the difference shown is not statistically significant

Despite the high donor chimerism observed in *Tert*^*−/−*^ recipients, a number of mice were euthanized before the planned four-month follow-up due to low body weight, and/or dehydration. NR significantly increased the number of G4 *Tert*^*−/−*^ mice that survived until the early endpoint collection (8 weeks post-transplant, Fig. 5a, b) and slightly improved survival of G3 *Tert*^*−/−*^ mice that survived until the final endpoint collection (16 weeks post-transplant, Fig. 5a, c). To determine the cause of death in G4 *Tert*^*−/−*^ mice, various tissues from vehicle- and NR-treated mice were submitted for histopathological analysis. No severe lesions were observed in the liver, lungs, or kidneys, in any of the mice. The cause of death in G4 *Tert*^*−/−*^ mice was likely due to enteritis, which was observed in all the mice (Fig. 5d) and is known to cause dehydration and malabsorption [51]. The severity of enteritis was drastically lessened in NR-treated, compared to the vehicle-treated G4 *Tert*^*−/−*^ mice (Fig. 5d). The small intestines of the vehicle-treated G4 *Tert*^*−/−*^ mice (Fig. 5d) showed mild to moderate villous atrophy, fusion, mucosal cell swelling, severe crypt hyperplasia, Paneth cell hyperplasia, areas of crypt loss, crypt degeneration, lamina propria fibrosis, and moderate lymphoplasmacytic infiltrates, in accordance with previous reports [23, 52]. The small intestines of the NR-treated G4 *Tert*^*−/−*^ mice also exhibited severe crypt hyperplasia, but displayed only mild lymphoplasmacytic infiltrates and villous atrophy (Fig. 5d, Table 1). Although no apparent differences in the severity of intestinal pathology were observed between vehicle-treated G1 and G3 *Tert*^*−/−*^ mice (Table 1), G4 *Tert*^*−/−*^ mice showed increased villous blunting and crypt hyperplasia compared to G1 and G3 *Tert*^*−/−*^ mice (Table 1). Altogether, these findings indicate that progressive telomere erosion exacerbates pathology of the small intestines post-irradiation, and NR improves the survival of G4 *Tert*^*−/−*^ mice in response to irradiation, presumably by ameliorating and/or delaying the onset of enteritis.

**Table 1:** NR alleviates inflammation and atrophy of the small intestine

Animal ID	Generation (*Tert*^−/−^)	Treatment	Villous blunting	Crypt hyperplasia	Paneth cell atrophy/loss	Crypt cell necrosis	Lamina propria infiltrates
4199	G4	Vehicle	2	4	3	2	3
4201	G4	Vehicle	3	4	2	3	3
4202	G4	NR	1	1	0	2–3	2
4198	G4	NR	2	4	2	2	2
4345	G3	Vehicle	1	3	3	3	3
4347	G3	NR	0	1	0	1	1–2
4183	G1	Vehicle	0	0	0	1	2
4029	G1	Vehicle	1	4	3	3	3
4024	G1	Vehicle	0	3	3	2	2
4181^#^	G1	Vehicle	2	2	0	2	2

Score: 0 (none), 1 (minimal), 2 (mild), 3 (moderate), 4 (severe). ^#^Tissue was mildly autolytic making proper evaluation difficult

## Discussion

Telomere attrition and NAD depletion are pivotal hallmarks of aging. Our recent findings unveiled the interconnection of these aging hallmarks and demonstrated benefits of boosting NAD with NR in human disease models of telomere dysfunction [43]. In this study, we sought to determine the effects of NAD supplementation on aspects of health span impaired by telomere erosion, including hematopoiesis, which is severely disrupted in human patients with TBDs. By utilizing late-generation *Tert*^*−/−*^ mice, we effectively demonstrated the benefits of boosting NAD on body weight, hematopoiesis, and resilience to DNA damage during conditions compromised by short telomeres.

Late-generation telomerase null mice exhibit decreased body weight with aging, which may arise from the concomitant villous atrophy of the small intestine [23–25, 52], a condition associated with malabsorption [53]. Interestingly, our results demonstrated that NR treatment helped prevent excess body weight loss in G3 *Tert*^*−/−*^ mice. Since enteritis can disrupt nutritional absorption [54], we speculate that the improvement in body weight by NR could be attributed to its beneficial effects on the small intestine. Our results showing that enteritis and villous atrophy were ameliorated by NR in transplanted late-generation *Tert*^*−/−*^ mice support this premise.

We did not detect significant alterations in the composition of stem/progenitor and blood cells within the bone marrow or blood cells in G3 *Tert*^*−/−*^ mice with and without NR treatment in a resting state (non-transplanted mice). However, we observed significant improvements with NR on B-lymphoid potential in G3 *Tert*^*−/−*^ mice in bone marrow transplantation experiments.

Transplant experiments utilizing late-generation *Tert*^*−/−*^ mice as recipients revealed striking benefits of NR on the micro/macroenvironment. NR increased donor B-lymphoid composition and decreased donor myeloid composition in blood from NR-treated G3 *Tert*^*−/−*^ recipients to frequencies more comparable to those observed in G1 *Tert*^*−/−*^ recipients. Furthermore, NR significantly decreased the number of neutrophils in the blood of G3 *Tert*^*−/−*^ transplant recipients, which were elevated compared to G1 *Tert*^*−/−*^ recipients. These findings support the use of NR to improve lymphoid potential and prevent myeloid skewing that occurs with aging [55, 56] and telomere attrition [22, 26]. Importantly, inflammatory cytokines, including the pro-inflammatory cytokines G-CSF, IL-1β, and IL-6, were elevated in the serum of G3 *Tert*^*−/−*^ transplant recipient mice compared to G1 *Tert*^*−/−*^ transplant recipients, suggesting that increased inflammation in the G3 *Tert*^*−/−*^ mice may have contributed to the myeloid skewing observed in G3 *Tert*^*−/−*^ mice. Evidence of this phenomenon is supported by previous studies [49, 57, 58]. For example, Pietras et al. observed an increased myeloid and decreased lymphoid output in mice injected intraperitoneally with IL-1β [49]. IL-6 and G-CSF are also known to promote myeloid differentiation of HSCs [58]. G-CSF is especially known for mobilizing hematopoietic stem and progenitor cells and driving the production and differentiation of neutrophils [50, 59]. Despite the increased myeloid composition observed in the blood of G3 compared to G1 *Tert*^*−/−*^ recipients, myeloid progenitors were decreased in the BM of vehicle-treated, but not NR-treated, G3 *Tert*^*−/−*^ compared to G1 *Tert*^*−/−*^ recipients. A possible explanation for this could be that G-CSF mobilized myeloid progenitors into the blood [60], leading to their depletion from the BM. Consistent with this notion as well as the decreased neutrophils observed in G3 *Tert*^*−/−*^ recipients upon NR treatment, NR also significantly decreased G-CSF expression in serum of G3 *Tert*^*−/−*^ recipients. These results are in agreement with a previous report showing increased G-CSF in the plasma of mice null of the telomerase RNA component (*Terc*^−/−^), compared to heterozygous *Terc*^+/−^ mice [26]. Consistently, *Terc*^*−/−*^ mice exhibited decreased B-lymphopoiesis and increased myelopoiesis.

Late-generation *Tert*^*−/−*^ recipient mice experienced decreased survival, and histopathological analysis revealed deficits of the small intestine as the likely cause. In mice and humans, telomerase is expressed and active in the small intestine, including intestinal stem cells (ISCs)[61–63]. In addition, *Tert*-expressing ISCs were previously found to contribute to crypt regeneration in response to injury induced by ionizing radiation [61]. Therefore, it is conceivable that *Tert* deficiency in the ISCs may have impeded regeneration in the small intestine of late-generation *Tert*^*−/−*^-irradiated mice. This effect also depends upon telomere length within the ISCs, as irradiated late-generation *Tert*^*−/−*^ mice showed reduced survival, compared to G1 *Tert*^*−/−*^ mice. Importantly, NR ameliorated the severity of villous atrophy, crypt degeneration, and enteritis in the small intestine and increased survival time in late-generation *Tert*^*−/−*^ mice. Correspondingly, NR has been found to alleviate aging-induced loss of ISCs and improve their resilience to dextran sulfate sodium-induced damage [64]. These findings are likely relevant to patients with telomere biology disorders with gastrointestinal manifestations including enteritis, enterocolitis, and malabsorption [8, 65].

Transplantation experiments using *Tert*^*−/−*^ mice not only elucidate the mechanism by which NR affects hematopoiesis, but also provide translational insight. Transplantation of late-generation *Tert*^*−/−*^ mice with healthy donor bone marrow is reminiscent of the setting by which human patients with telomere biology disorders undergo stem cell transplantation. NR treatment has been shown to increase NAD and exert anti-inflammatory effects in humans [46, 66]. In agreement, our results demonstrate a beneficial anti-inflammatory effect of NR on the host environment, which may facilitate production of a more balanced lineage/increased lymphoid composition by donor stem cells. Our previous study demonstrated that NR mitigated NAD deficiency and consequently the imbalanced NAD-consuming enzymatic activities, which alleviated mitochondrial impairment, telomere dysfunction, and the production of pro-inflammatory cytokines in primary fibroblasts from patients with DC [32]. As NR can boost NAD levels in late-generation *Tert*^*−/−*^ mice, it may restore balance of NAD-consuming enzymes in the BM and/or PB, such as increasing SIRT1 activation, thereby suppressing production of pro-inflammatory cytokines [67], improving mitochondrial function [42], and/or reducing senescence. Thus, it is possible that improved mitochondrial function or reduced senescence could account for decreased release of pro-inflammatory cytokines driven by oxidative stress or a senescence-associated secretory phenotype in BM and/or PB of *Tert*^*−/−*^ mice. Collectively, our results demonstrating improved B-lymphoid potential and telomere integrity, decreased serum expression of G-CSF, as well as mitigation of systemic inflammation and irradiation-induced pathology of the small intestine in late-generation *Tert*^*−/−*^ mice support the use of NAD supplementation with NR to ameliorate deleterious conditions associated with or induced by telomere erosion. Our findings illuminate mechanisms underlying the benefits of NR in the context of telomere erosion and advance progress in developing therapeutic strategies for telomere biology disorders and potentially for humans with a variety of stresses and conditions that are associated with telomere shortening [68].

### Supplementary Information

Below is the link to the electronic supplementary material.

Fig. S1 NR treatment does not affect hematopoietic stem/progenitor or blood cell composition in non-transplanted mice. **a-b)** Bar graphs show the frequency of live common lymphoid progenitors (CLP, Lin-IL-7Rα+Flk2+), myeloid progenitors (MyProg, Lineage-cKit+), and HSCs (Lineage-Sca1+CD34-Flk2-) in **a)** G1 vehicle-treated (blue, n=2), G3 vehicle-treated (n=4, red), and G3 NR-treated (n=2, gray) mice treated for 2 months and **b)** G3 vehicle-treated (n=4, red), and G3 NR-treated (n=4, gray) mice treated for 10 months. **c)** Intracellular NAD+ levels in bone marrow derived from G1 (n=3) or G3 (n=3) *Tert*^-/-^ mice. **d)** Flow cytometry analysis showing the lineage contribution of B cells (B220^+^, blue), myeloid cells (Mac1^+^, red), and T cells (CD3^+^, gray) in vehicle- (n=4) and NR-treated (n=2) G3 *Tert*^-/-^ mice. **e)** Representative immunofluorescent images stained with anti-Ki67 alone (green, top) and merged with DAPI (blue, bottom) in isolated HSCs from vehicle-treated G1 (n=1), vehicle-treated G3 (n=3), and NR-treated G3 (n=2) *Tert*^-/-^ mice after 2 months treatment. Scale bars: 10μm. **f)** Bar graph shows the percentage of Ki-67 positive cells in each image. Statistical analyses were performed using two-way ANOVAs in **a-b,d,** a student’s unpaired t-test in **c,** and a one-way ANOVA in **f**. Data are not statistically significant. Data are mean ± SEM. (PNG 285 kb)

High resolution image (TIF 11924 kb)

Fig. S2 NR treatment does not affect hematopoietic stem/progenitor frequencies in transplant recipients of *Tert*^-/-^ donor BM. **a)** *Tert*^-/-^ donor derived bone marrow (BM) chimerism, and **b)** the frequency of *Tert*^-/-^ donor derived HSCs (Lineage-Sca1+CD34-Flk2-), myeloid progenitors (MyProg, Lineage-cKit+Sca1-), and common lymphoid progenitors (CLP, Lin-CD27+IL-7Rα+Flk2+). Statistical analysis was performed using a student’s unpaired *t*-tests (a) and a two-way ANOVA (b). Data are mean ± SEM. n=10 mice per group with the exception of few samples (3) with insufficient cell numbers due to technical errors in the HSC/MyProg panels. The p values are not significant. (PNG 57 kb)

High resolution image (TIF 3152 kb)

Fig. S3 NR improves telomere integrity in recipients of *Tert*^-/-^ donor BM. **a)** Representative TIFs showing colocalization of telomere DNA (red) and 53BP1 (green) in whole bone marrow cells derived from primary bone marrow transplant recipients of G3 vehicle or NR-treated *Tert*^-/-^ mice by IF-Telomere FISH analysis. White frames: regions for enlarged view at right panels. Scale bars, 10 μm. **b)** Quantification of the number of TIFs per cell derived from bone marrow recipients of G3 vehicle- or NR-treated mice. n=2 mice in each group. ~100 cells/group were counted. Data points represent individual cells. **c)** Quantification of the percentage of bone marrow cells with ≥5 TIFs in each mouse. Data points represent individual mice. The p values were determined by a student’s unpaired *t*-test in b and by a Fisher’s exact test in c. Data are mean ± SEM. (PNG 182 kb)

High resolution image (TIF 3591 kb)

Fig. S4 NR prevents a decreased frequency of myeloid progenitors in BM of *Tert*^-/-^ recipients. **a)** Vehicle and NR-treated *CD45.1* donor chimerism of peripheral blood in transplant recipients at 4-16 weeks post-transplant. **b)** The frequency of donor (CD45.1) derived HSC (Lineage- Sca1+CD34-Flk2-), myeloid progenitors (MyProg, Lineage-cKit+Sca1-), and common lymphoid progenitors (CLP, Lin-CD27+IL-7Rα+Flk2+). Mice were treated with vehicle or NR for 1 month prior to transplantation. n=3, 4, 4 for G1 veh, G3 veh, and G3 NR-treated mice, respectively. The p-values were determined by two-way ANOVAs with Tukey’s multiple comparisons. Data are mean ± SEM. (PNG 64 kb)

High resolution image (TIF 3605 kb)

Supplementary Table 1 (XLSX 36 kb)

Supplementary Table 2 (XLSX 23 kb)

## Data Availability

The authors declare that all data supporting the findings of this study are available within the paper and its supplementary information files.
