# Supplementary figures and images for: Boosting NAD ameliorates hematopoietic impairment linked to short telomeres in vivo
Source: GeroScience. 2023 Feb 24;45(4):2213–28. doi: 10.1007/s11357-023-00752-2 (PMC10651621; doi:10.1007/s11357-023-00752-2)

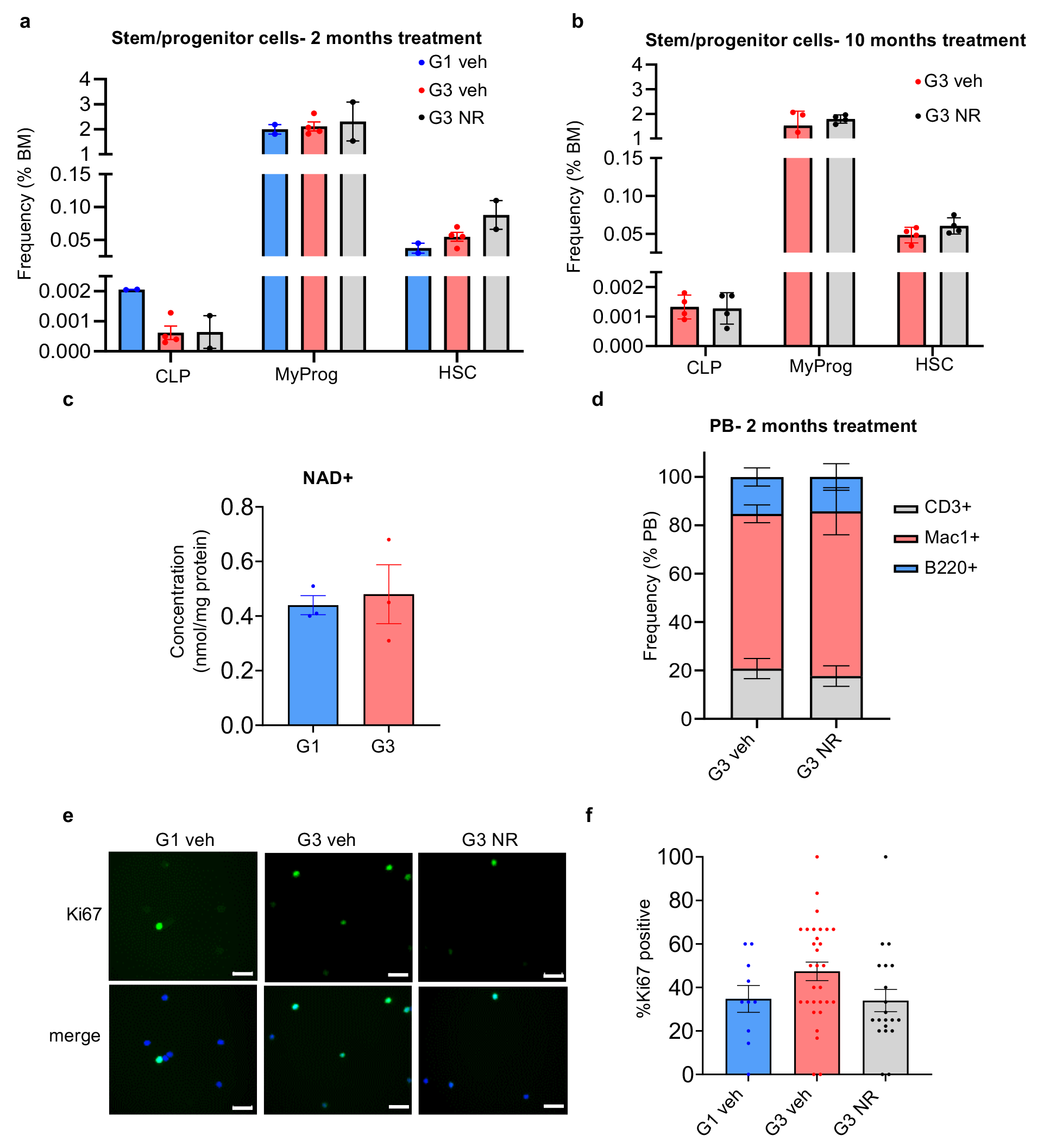

Supplement: Supplementary file 1 — NR treatment does not affect hematopoietic stem/progenitor or blood cell composition in non-transplanted mice. a-b) Bar graphs show the frequency of live common lymphoid progenitors (CLP, Lin-IL-7Rα+Flk2+), myeloid progenitors (MyProg, Lineage-cKit+), and HSCs (Lineage-Sca1+CD34-Flk2-) in a) G1 vehicle-treated (blue, n=2), G3 vehicle-treated (n=4, red), and G3 NR-treated (n=2, gray) mice treated for 2 months and b) G3 vehicle-treated (n=4, red), and G3 NR-treated (n=4, gray) mice treated for 10 months. c) Intracellular NAD+ levels in bone marrow derived from G1 (n=3) or G3 (n=3) Tert-/- mice. d) Flow cytometry analysis showing the lineage contribution of B cells (B220+, blue), myeloid cells (Mac1+, red), and T cells (CD3+, gray) in vehicle- (n=4) and NR-treated (n=2) G3 Tert-/- mice. e) Representative immunofluorescent images stained with anti-Ki67 alone (green, top) and merged with DAPI (blue, bottom) in isolated HSCs from vehicle-treated G1 (n=1), vehicle-treated G3 (n=3), and NR-treated G3 (n=2) Tert-/- mice after 2 months treatment. Scale bars: 10μm. f) Bar graph shows the percentage of Ki-67 positive cells in each image. Statistical analyses were performed using two-way ANOVAs in a-b,d, a student’s unpaired t-test in c, and a one-way ANOVA in f. Data are not statistically significant. Data are mean ± SEM. (PNG 285 kb) [file 11357_2023_752_Fig6_ESM.png]

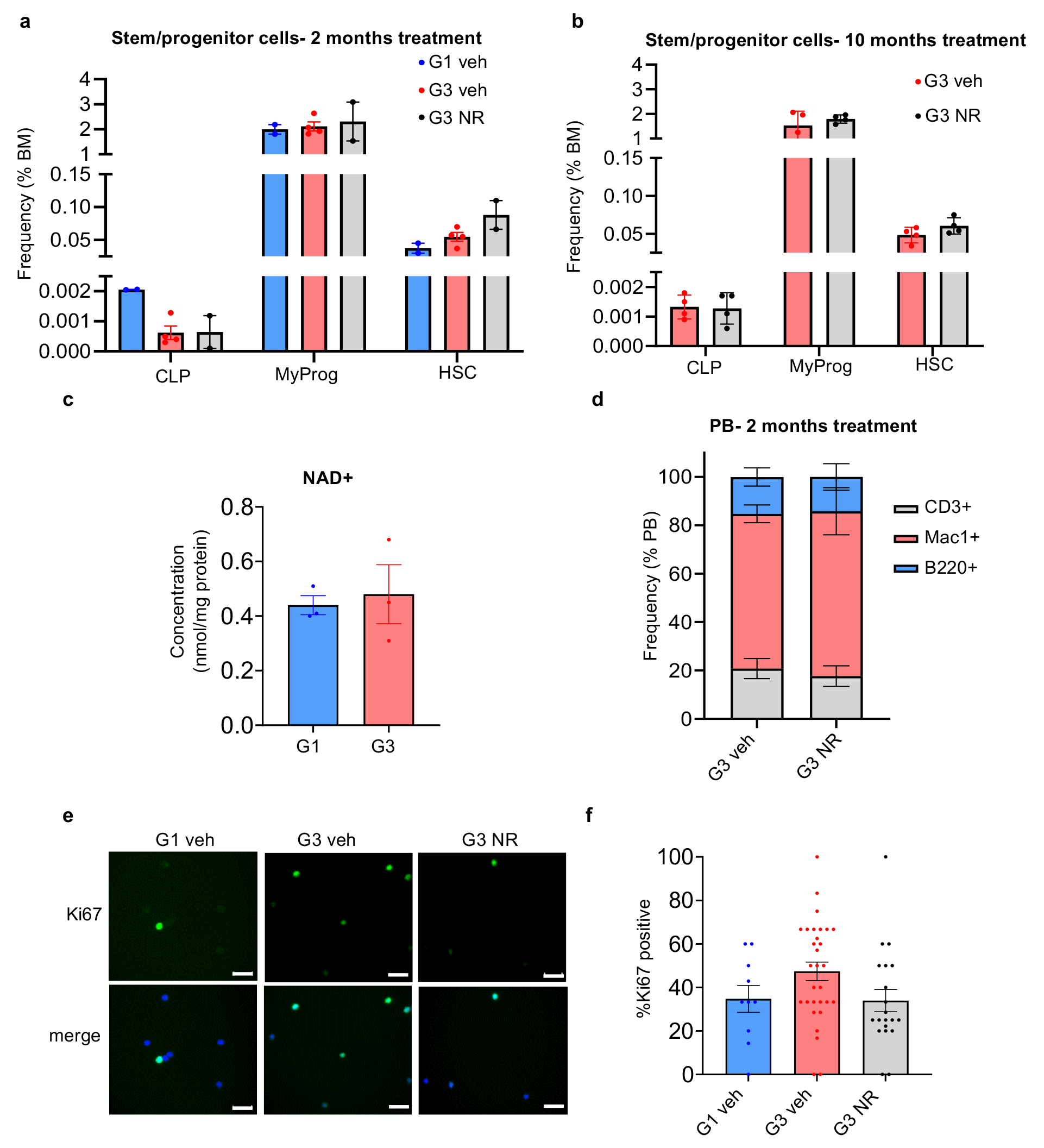

Supplement: Supplementary file 2 — High resolution image (TIF 11924 kb) [file 11357_2023_752_MOESM1_ESM.tif]

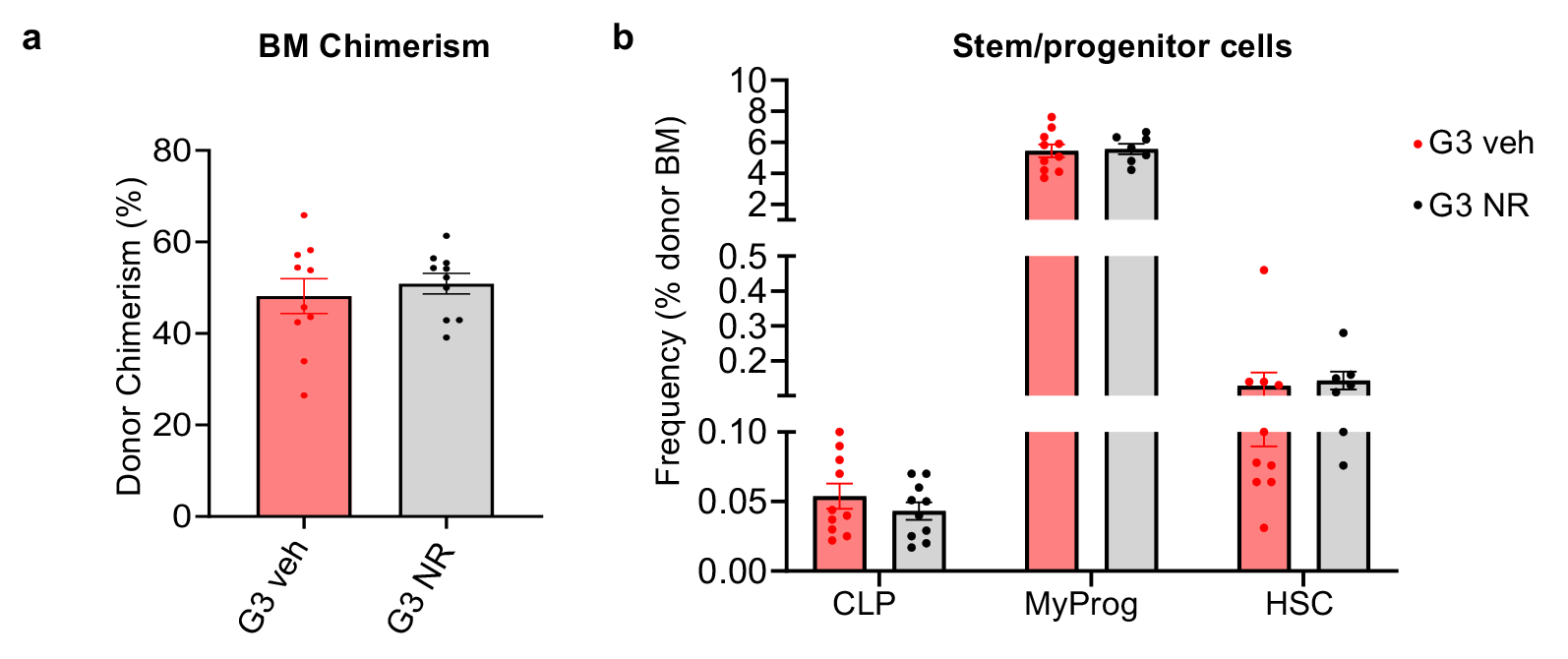

Supplement: Supplementary file 3 — NR treatment does not affect hematopoietic stem/progenitor frequencies in transplant recipients of Tert-/- donor BM. a) Tert-/- donor derived bone marrow (BM) chimerism, and b) the frequency of Tert-/- donor derived HSCs (Lineage-Sca1+CD34-Flk2-), myeloid progenitors (MyProg, Lineage-cKit+Sca1-), and common lymphoid progenitors (CLP, Lin-CD27+IL-7Rα+Flk2+). Statistical analysis was performed using a student’s unpaired t-tests (a) and a two-way ANOVA (b). Data are mean ± SEM. n=10 mice per group with the exception of few samples (3) with insufficient cell numbers due to technical errors in the HSC/MyProg panels. The p values are not significant. (PNG 57 kb) [file 11357_2023_752_Fig7_ESM.png]

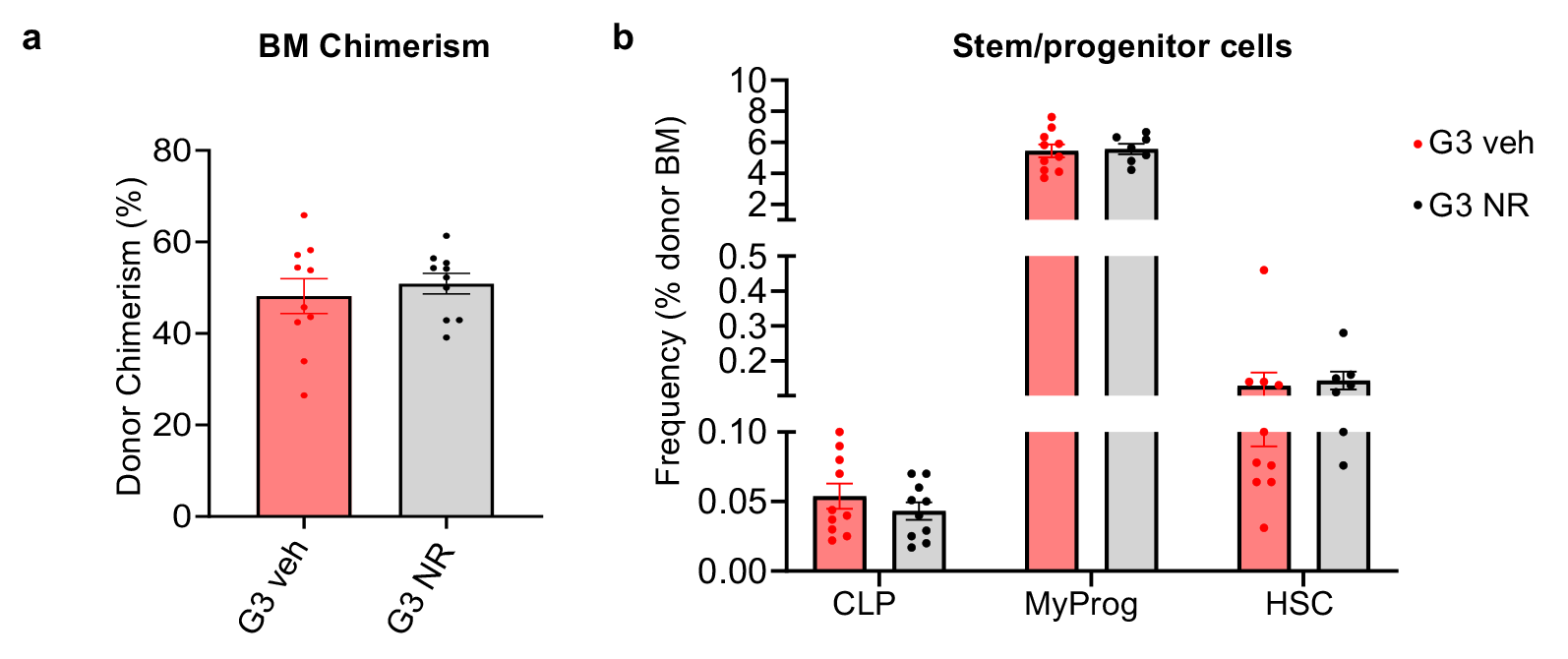

Supplement: Supplementary file 4 — High resolution image (TIF 3152 kb) [file 11357_2023_752_MOESM2_ESM.tif]

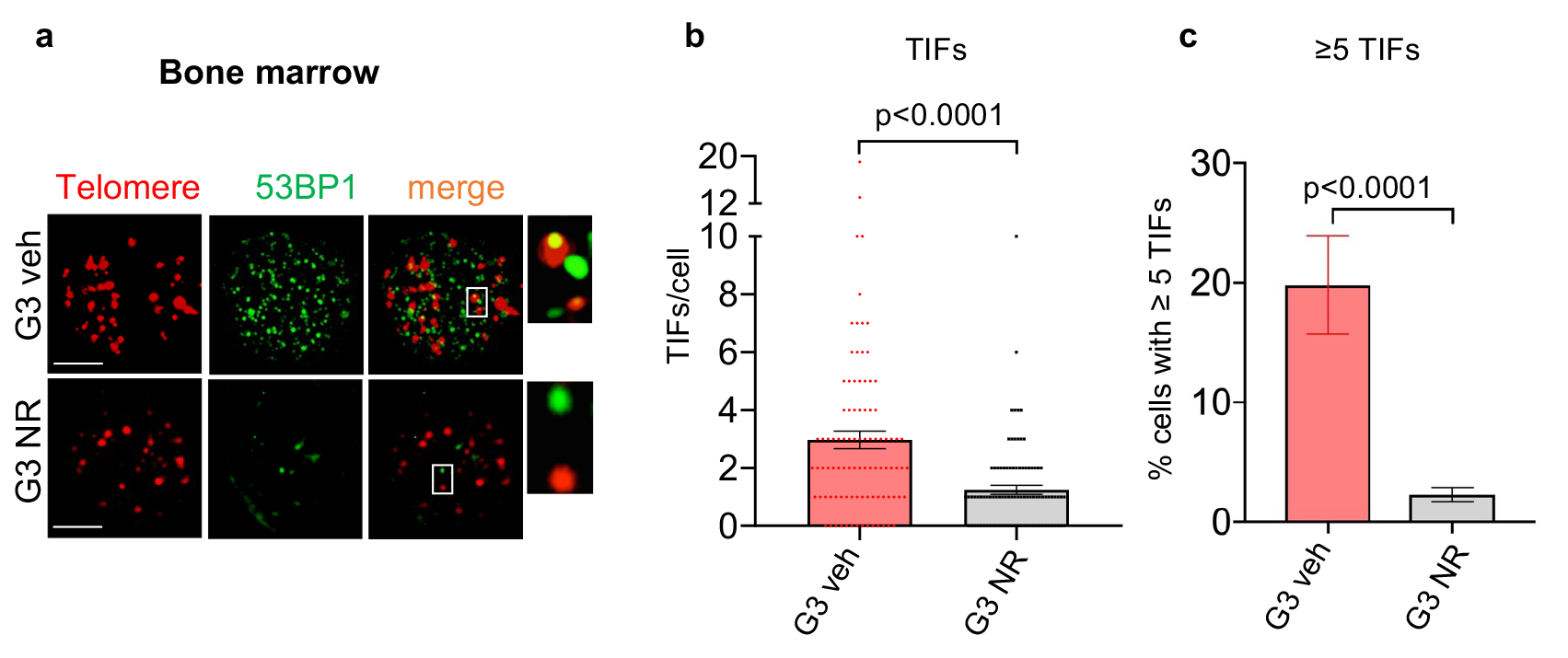

Supplement: Supplementary file 5 — NR improves telomere integrity in recipients of Tert-/- donor BM. a) Representative TIFs showing colocalization of telomere DNA (red) and 53BP1 (green) in whole bone marrow cells derived from primary bone marrow transplant recipients of G3 vehicle or NR-treated Tert-/- mice by IF-Telomere FISH analysis. White frames: regions for enlarged view at right panels. Scale bars, 10 μm. b) Quantification of the number of TIFs per cell derived from bone marrow recipients of G3 vehicle- or NR-treated mice. n=2 mice in each group. ~100 cells/group were counted. Data points represent individual cells. c) Quantification of the percentage of bone marrow cells with ≥5 TIFs in each mouse. Data points represent individual mice. The p values were determined by a student’s unpaired t-test in b and by a Fisher’s exact test in c. Data are mean ± SEM. (PNG 182 kb) [file 11357_2023_752_Fig8_ESM.png]

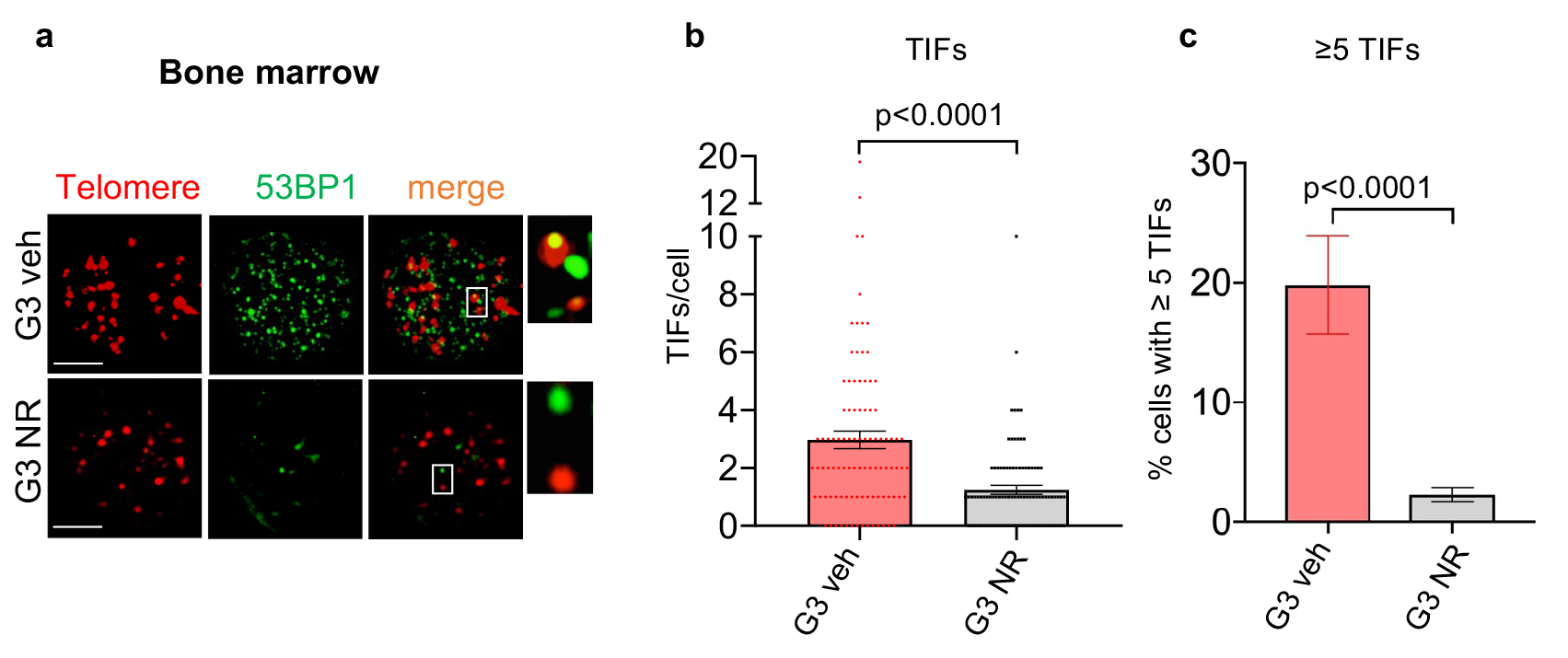

Supplement: Supplementary file 6 — High resolution image (TIF 3591 kb) [file 11357_2023_752_MOESM3_ESM.tif]

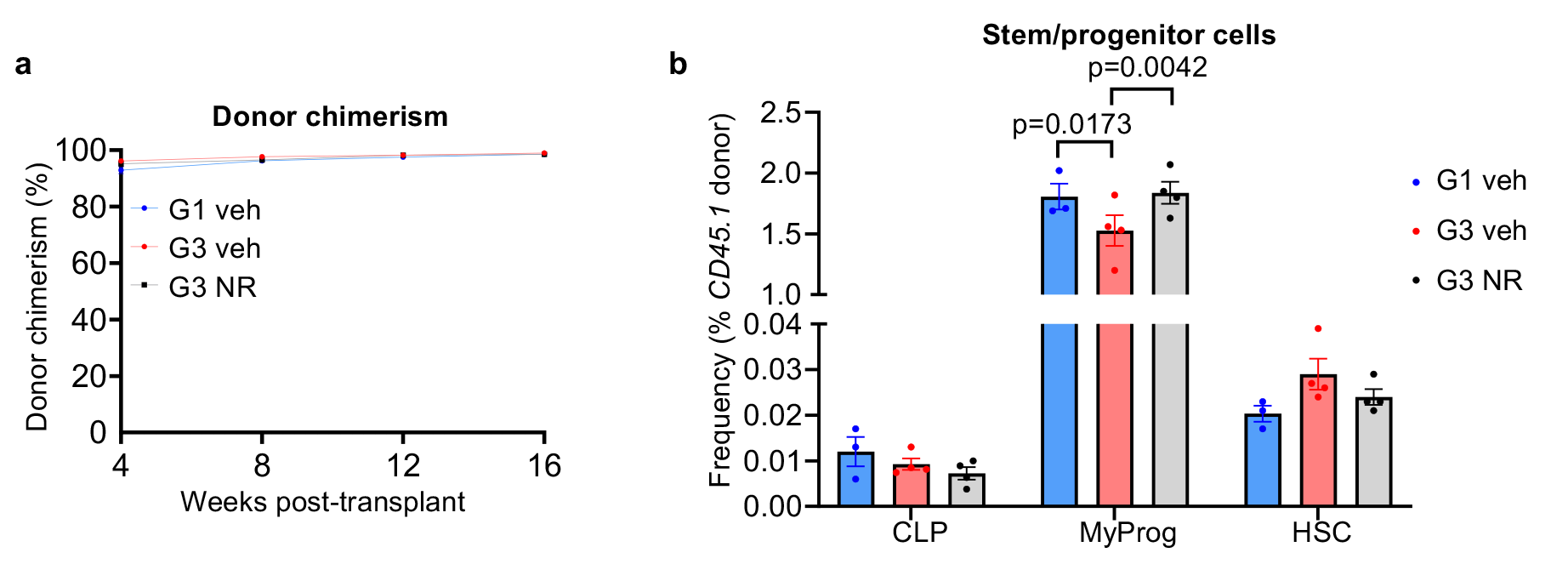

Supplement: Supplementary file 7 — NR prevents a decreased frequency of myeloid progenitors in BM of Tert-/- recipients. a) Vehicle and NR-treated CD45.1 donor chimerism of peripheral blood in transplant recipients at 4-16 weeks post-transplant. b) The frequency of donor (CD45.1) derived HSC (Lineage- Sca1+CD34-Flk2-), myeloid progenitors (MyProg, Lineage-cKit+Sca1-), and common lymphoid progenitors (CLP, Lin-CD27+IL-7Rα+Flk2+). Mice were treated with vehicle or NR for 1 month prior to transplantation. n=3, 4, 4 for G1 veh, G3 veh, and G3 NR-treated mice, respectively. The p-values were determined by two-way ANOVAs with Tukey’s multiple comparisons. Data are mean ± SEM. (PNG 64 kb) [file 11357_2023_752_Fig9_ESM.png]

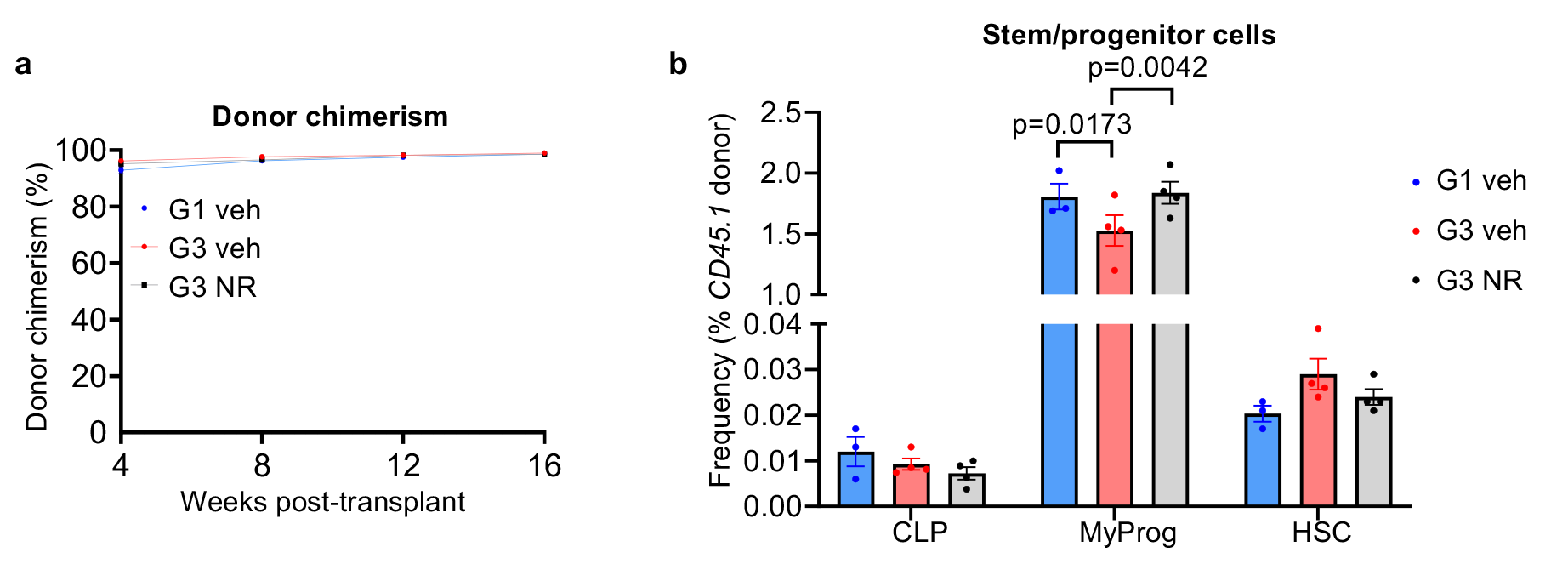

Supplement: Supplementary file 8 — High resolution image (TIF 3605 kb) [file 11357_2023_752_MOESM4_ESM.tif]
